# Association between Plasma Nonesterified Fatty Acids Species and Adipose Tissue Fatty Acid Composition

**DOI:** 10.1371/journal.pone.0074927

**Published:** 2013-10-02

**Authors:** Christian Hellmuth, Hans Demmelmair, Isabel Schmitt, Wolfgang Peissner, Matthias Blüher, Berthold Koletzko

**Affiliations:** 1 Division of Metabolic and Nutritional Medicine, Dr. von Hauner Children's Hospital, University of Munich Medical Center, Munich, Germany; 2 Department for Internal Medicine, Clinic for Endocrinology and Nephrology, University Hospital Leipzig, Leipzig, Germany; University College Dublin, Ireland

## Abstract

Fatty acid composition of adipose tissue (AT) is an established long-term biomarker for fatty acid (FA) intake and status, but AT samples are not easily available. Nonesterified FA composition in plasma (pNEFA) may be a good indicator of AT FA composition, because pNEFA are mainly generated by AT lipolysis. We investigated the correlation of 42 pNEFA and subcutaneous as well as visceral AT FA in 27 non-diabetic women with a median BMI of 36 kg/m^2^ (Q_0.25_: 25 kg/m^2^; Q_0.75_: 49 kg/m^2^). Close correlations of pNEFA and AT FA were found for odd-chain FA (15∶0 r = 0.838 and 0.862 for subcutaneous and visceral AT, respectively) and omega-3 FA (22∶6 r = 0.719/0.535), while no significant or low correlations were found for other FA including 18∶1 (r = 0.384/0.325) and 20∶4 (r = 0.386/0.266). Close correlations of pNEFA and AT FA were found for essential fatty acids, like 18∶2 (r = 0.541/0.610) and 20∶5 (r = 0.561/0.543). The lower correlation for some pNEFA species with AT FA indicates that the variation of most pNEFA is significantly affected by other FA sources and flux of FA to tissue, in addition to release from AT. A relevant influence of BMI on the level of correlation was shown for saturated FA. NEFA analysis in fasted plasma can serve as a virtual AT biopsy for some FA, and as a biomarker for intake of dairy products and sea fish.

## Introduction

Fatty acid (FA) composition of adipose tissue (AT) is a well-accepted biomarker for the assessment of long-term dietary FA intake, considered to be superior to dietary records and food frequency questionnaires [1]. Percentage contributions of FA in AT, representing intake of dairy products, fish or fish oil are highly correlated to dietary intake [2], [3]. While essential polyunsaturated FA (PUFA) show a close relationship between dietary intake and AT content, saturated FA (SFA) and monounsaturated FA (MUFA) are less closely correlated [4], presumably because these FA are derived from both diet and endogenous synthesis [5]. Nevertheless, SFA and MUFA in AT are of importance as biomarkers for various disease risks [3]. Alterations of AT fatty acid composition appear to play a crucial role in the development of insulin resistance and diabetes [6], [7], [8].

Although AT is a biomarker for the intake of FA and reflects FA metabolism, routine determination of AT composition is not practical due to the invasive nature of sample collection via biopsies, particularly in larger clinical trials or in vulnerable populations such as children. During fasting, AT lipolysis releases nonesterified fatty acids into plasma (pNEFA). Thus, pNEFA could provide a valuable surrogate marker for AT FA composition. Some studies indicate a close correlation between pNEFA and AT FA content for some FA [1]. To our knowledge, only Yli-Jama et al. investigated the relationship of AT and pNEFA for a large number of 27 FA in a sizable group of patients with myocardial infarction and of controls [9]. They reported widely differing coefficients of correlation between AT and pNEFA for individual FA.

So far, most studies of the relationship between pNEFA and AT focused on specific metabolic steps, e.g. mobilization [10] or (re-)uptake of pNEFA by adipocytes [11]. Mobilization studies were mostly performed in-vitro [12], in animals with induced lipolysis [13] or using venous-arterial differences of human AT, which indicated a preferential mobilization of PUFA [14], [15]. These approaches do not reflect the relation of AT and pNEFA FA percentages, because the pNEFA pool is affected by a complex interaction of AT lipolysis [10], reincorporation of NEFA into AT triacylglycerols (TAG) [16], uptake of pNEFA by peripheral tissues, oxidation rates of individual FA [17], intracellular metabolism [18] and contribution of pNEFA derived from plasma TAG or phospholipid hydrolysis [19].

Since there is limited information on relationship between pNEFA and AT FA composition, the objective of this study is to explore the relationship of fatty acid composition of pNEFA, visceral AT and subcutaneous AT in subjects with differing BMI. We used a sensitive and precise LC-MS/MS method [20] enabling the quantification of more than 40 FA, including very long-chain fatty acids (VLCFA), saturated and unsaturated odd-chain fatty acids and C24 intermediates of the endogenous n-3 docosahexaenoic acid and n-6 docosapentaenoic acid synthesis.

## Materials and Methods

### Ethics Statement

The study protocol was approved by the Ethical Committee of the University of Leipzig Medical Center. Written informed consent was obtained from all subjects.

### Subjects

Participants were recruited at the University of Leipzig (Department for Internal Medicine, Division for Endocrinology and Nephrology). Fatty acid composition was investigated in 27 donors of paired visceral omental (vAT) and subcutaneous abdominal adipose tissue (sAT) samples, who underwent abdominal surgery for weight reduction (sleeve gastrectomy or Roux en Y gastric bypass), cholecystectomy, or explorative laparotomy. All subjects had a stable weight, defined as the absence of fluctuations of >2% of body weight for at least 3 months before surgery. Intake of any medication affecting glucose and lipid metabolism were defined as exclusion criteria. Adipose tissue was taken during surgery and immediately frozen in liquid nitrogen. Plasma samples were taken after prolonged fasting of more than 12 h on the day of surgery before anaesthetization. Blood samples were collected into refrigerated EDTA containing tubes and centrifuged; subsequently the plasma was aliquoted and stored at −80°C.

All subjects were Caucasian, female and non-diabetic with a mean age of 55±14 years (M±SD). Diabetes was excluded by fasted glucose and HbA1c analysis. The BMI of the study participants was not normally distributed and ranged from normal weight to extremely obese with a median BMI of 36 kg/m^2^ (Q_0.25_: 25 kg/m^2^; Q_0.75_: 49 kg/m^2^).The group consisted of 8 normal-weighted (BMI<25 kg/m^2^, age: 60±15 years), 4 over-weighted (BMI: 25 to 30 kg/m^2^, age: 64±9 years), 7 obese (BMI: 30 to 40 kg/m^2^, age: 56±15 years) and 8 morbidly obese (BMI>40 kg/m^2^, age: 44±9 years) patients.

### Methods

All used chemicals were obtained in highest purity available (suppliers: Merck, Darmstadt, Germany; Promochem, Wesel, Germany; Fluka Sigma-Aldrich Chemistry GmbH, Steinheim, Germany).

### Plasma samples

Sample preparation for pNEFA analysis was performed as previously reported [20]. Briefly, 20 µl of plasma were mixed with 200 µl isopropanol (containing 2 mg/100 ml uniformly ^13^C-labelled palmitic acid) in a 96-deepwell plate. After centrifugation the supernatant was transferred into a 96-well plate for LC-MS/MS analysis.

### Adipose tissue samples

Homogenisation of adipose tissue samples (about 50 mg) was performed in 2 ml Biozym Cryovials (Biozym Scientific GmbH, Oldendorf, Germany) containing glass pellets and 1000 µl chloroform∶methanol (CHCl_3_∶MeOH 2∶1, +5 g/l butylated hydroxytoluene) for 2×30 seconds at 7134× g with a MagNA Lyser Instrument (Roche Diagnostics, Mannheim, Germany).

After cell lysis the vials were centrifuged for 10 min at 2330× g (room temperature). 10 µl of the supernatant were used for hydrolysis of total lipids according to Pettinella et al. [21]. The aliquot was dissolved in 850 µl CHCl_3_∶MeOH (1∶8) and after the addition of 150 µl KOH (40%) hydrolysis was performed in a nitrogen atmosphere at 60°C for 30 min. After cooling to room temperature, 700 µl phosphate buffer (pH = 7.4, 50 mM) and 300 µl HCl (2.5 mM) were added. Extraction was performed with 2×2000 µl hexane∶diethylether (1∶1, v/v). The upper layer was taken off, dried under nitrogen flow and FA redissolved in 500 µl isopropanol for LC/MS-MS.

In agreement with previous observations [1], [22], separation of dissolved AT lipids by TLC and subsequent quantification by gas chromatography revealed that TAG contributed more than 97% to total lipids. Therefore, extracts were directly hydrolysed without further purification.

### Liquid Chromatography and ESI-MS/MS

LC-MS/MS analysis was performed as previously reported [20]. Briefly, an UPLC diphenyl column (Pursuit UPS Diphenyl, 1.9 µm, 100 mm×3.0 mm; Varian, Darmstadt, Germany) was used for chromatographic separation at 40°C with an Agilent 1200 SL series HPLC system (Waldbronn, Germany). The injection volume was set to 10 µL for plasma samples and to 2 µl for AT samples with an eluent flow rate of 700 µL/min. Equilibration was performed for 2.5 min with 45% of eluent A (water containing 5 mM ammonium acetate, 2.1 mM acetic acid) and 55% of eluent B (acetonitrile with 20% isopropanol), eluent B was linearly increased to 95% at a duration of 4 min and was held constant for 3 min.

A hybrid triple quadrupole mass spectrometer (4000 QTRAP, AB Sciex, Darmstadt, Germany) operating in negative ESI mode was coupled to the HPLC system [20]. Collision energy was optimized for each fatty acid individually to obtain signals well above the quantification limit, but within the linear response range.

With the analytical method applied, fatty acids are separated according to chain length and number of double bonds, but not according to position of double bonds.

### Statistical analysis

Data analysis was performed with Microsoft Excel 2010 (Microsoft Inc., Redmond, WA) and “R: A language and environment for statistical computing” [23]. FA levels in pNEFA, sAT and vAT are presented as molar percentages of total FA analysed unless explicitly stated otherwise. Most fatty acid percentages were not normally distributed according to histograms, QQ plots and Anderson-Darling tests, results are given as median and interquartile ranges. Spearman's rho statistics were used to estimate rank-based correlation coefficients (r). Since the p-value does not reflect the strength of the relationship but strongly depends on sample number, p-values for correlation analyses are only mentioned in the corresponding figure and not substance for the interpretation of results. Wilcoxon signed rank tests were performed with a limit for statistical significance of <0.001068 due to multiple testing. The significance limit was calculated according to the Sidak correction [24]:


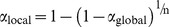
(1)

With a global significance level α_global_ of 0.05 for n = 48 (42 FA+6 sum parameters).

To evaluate influence of BMI on the correlation of AT and pNEFA, two quantile regression models were implemented to describe variance of AT FA percentages with pNEFA only or with BMI and pNEFA using the R package “quantreg” [25]. The two models were compared by calculated R-squared (r^2^) and ANOVA.

To predict the FA percentages of 15∶0, 17∶0 and 22∶6 which were strongly correlated between compartments and were normally distributed in the 3 compartments, the “glm” function with multiple linear regression (MLR) and leave-one-out cross-validation (CV) of the R package “boot” was utilized [26]. Model 1 was established using pNEFA and BMI for AT FA prediction, while model 2 used pNEFA only.

The model comparisons were performed with ANOVA. Absolute prediction error and adjusted r^2^ were estimated to compare different models. Absolute prediction error for every FA was calculated as the mean absolute differences of analysed and predicted values after CV validation.

## Results

42 fatty acids were analysed in sAT, vAT and plasma samples with good precision (CV<20%).

### Fatty acid composition

Both adipose tissues and pNEFA contained high concentrations of MUFA, dominated by 18∶1, followed by SFA and PUFA (Table1).

**Table 1:** Fatty acid composition of plasma nonesterified fatty acids (pNEFA), subcutaneous adipose tissue (sAT) and visceral adipose tissue (vAT).

FA	pNEFA	sAT	vAT
**12∶0** *	0.312% [0.263%,0.450%]	1.076% [0.812%,1.402%]	1.258% [1.027%,1.642%]
**12∶1** *	0.065% [0.051%,0.083%]	0.207% [0.137%,0.251%]	0.236% [0.206%,0.339%]
**14∶0**	1.934% [1.710%,2.198%]	5.442% [4.894%,6.646%]	6.020% [5.134%,6.447%]
**14∶1**	0.226% [0.181%,0.334%]	0.785% [0.681%,0.862%]	0.837% [0.742%,0.924%]
**15∶0**	0.400% [0.339%,0.519%]	0.846% [0.673%,0.963%]	0.808% [0.675%,0.956%]
**16∶0**	26.659% [24.985%,28.172%]	22.152% [21.326%,22.797%]	21.548% [21.029%,22.588%]
**16∶1**	5.504% [4.584%,6.559%]	8.740% [7.281%,10.514%]	9.378% [7.895%,10.089%]
**16∶2**	0.050% [0.040%,0.060%]	0.109% [0.100%,0.122%]	0.110% [0.100%,0.121%]
**17∶0**	0.445% [0.403%,0.503%]	0.545% [0.469%,0.609%]	0.528% [0.427%,0.625%]
**17∶1**	0.383% [0.347%,0.407%]	0.459% [0.434%,0.508%]	0.471% [0.438%,0.493%]
**17∶2**	0.009% [0.008%,0.012%]	0.021% [0.020%,0.023%]	0.021% [0.019%,0.023%]
**18∶0**	7.819% [6.967%,8.823%]	5.264% [4.478%,6.403%]	5.428% [4.706%,6.108%]
**18∶1**	40.367% [39.445%,41.434%]	36.294% [35.178%,36.809%]	35.949% [35.556%,36.500%]
**18∶2**	9.959% [9.333%,11.329%]	10.649% [9.791%,10.874%]	10.437% [9.543%,11.006%]
**18∶3**	1.392% [1.148%,1.769%]	1.717% [1.567%,2.021%]	1.683% [1.425%,1.968%]
**18∶4**	0.017% [0.013%,0.022%]	0.038% [0.033%,0.047%]	0.044% [0.039%,0.056%]
**19∶0**	0.040% [0.037%,0.051%]	0.05% [0.041%,0.059%]	0.062% [0.045%,0.071%]
**19∶1**	0.166% [0.149%,0.193%]	0.263% [0.223%,0.296%]	0.277% [0.231%,0.302%]
**19∶2**	0.014% [0.012%,0.016%]	0.021% [0.019%,0.022%]	0.019% [0.018%,0.022%]
**20∶0** *	0.044% [0.038%,0.058%]	0.193% [0.152%,0.276%]	0.258% [0.240%,0.350%]
**20∶1**	0.478% [0.409%,0.509%]	1.364% [1.125%,1.556%]	1.399% [1.230%,1.583%]
**20∶2+**	0.220% [0.197%,0.231%]	0.500% [0.452%,0.533%]	0.444% [0.400%,0.512%]
**20∶3+**	0.230% [0.205%,0.262%]	0.576% [0.444%,0.629%]	0.396% [0.358%,0.511%]
**20∶4+**	0.682% [0.534%,0.729%]	0.868% [0.703%,0.907%]	0.654% [0.557%,0.714%]
**20∶5+**	0.107% [0.078%,0.160%]	0.178% [0.120%,0.218%]	0.130% [0.094%,0.161%]
**22∶0** *	0.015% [0.011%,0.023%]	0.021% [0.012%,0.031%]	0.033% [0.028%,0.048%]
**22∶1** *	0.042% [0.025%,0.049%]	0.129% [0.076%,0.186%]	0.193% [0.127%,0.253%]
**22∶2** *	0.008% [0.006%,0.009%]	0.015% [0.012%,0.018%]	0.018% [0.016%,0.020%]
**22∶3**	0.010% [0.009%,0.013%]	0.030% [0.023%,0.039%]	0.031% [0.024%,0.039%]
**22∶4+**	0.124% [0.108%,0.134%]	0.361% [0.250%,0.395%]	0.266% [0.225%,0.316%]
**22∶5+**	0.222% [0.189%,0.254%]	0.450% [0.362%,0.505%]	0.350% [0.295%,0.405%]
**22∶6**	0.498% [0.350%,0.598%]	0.392% [0.285%,0.458%]	0.317% [0.234%,0.386%]
**24∶0** *	0.020% [0.016%,0.027%]	0.012% [0.008%,0.019%]	0.021% [0.015%,0.029%]
**24∶1** *	0.045% [0.037%,0.054%]	0.016% [0.011%,0.031%]	0.031% [0.021%,0.045%]
**24∶2** *	0.005% [0.004%,0.006%]	0.003% [0.002%,0.004%]	0.004% [0.003%,0.006%]
**24∶3** *	0.001% [0.001%,0.001%]	0.001% [0.000%,0.001%]	0.001% [0.001%,0.001%]
**24∶4** *	0.005% [0.004%,0.006%]	0.003% [0.002%,0.003%]	0.004% [0.003%,0.005%]
**24∶5** *	0.006% [0.005%,0.007%]	0.008% [0.006%,0.009%]	0.010% [0.007%,0.013%]
**24∶6** *	0.004% [0.003%,0.005%]	0.006% [0.005%,0.009%]	0.008% [0.006%,0.011%]
**26∶0** *	0.002% [0.001%,0.003%]	0.003% [0.002%,0.004%]	0.005% [0.003%,0.006%]
**26∶1** *	0.003% [0.003%,0.005%]	0.002% [0.001%,0.004%]	0.003% [0.002%,0.006%]
**26∶2** *	0.002% [0.002%,0.002%]	0.001% [0.001%,0.001%]	0.001% [0.001%,0.002%]
**SFA**	37.559% [35.820%,39.735%]	36.429% [33.455%,38.296%]	36.591% [34.518%,38.109%]
**MUFA**	47.405% [46.673%,49.373%]	48.300% [46.207%,49.412%]	48.638% [47.261%,50.094%]
**PUFA+**	13.697% [12.640%,15.359%]	15.952% [14.694%,16.552%]	14.804% [13.923%,16.606%]
**n3+**	2.237% [1.898%,2.656%]	2.812% [2.618%,3.048%]	2.593% [2.448%,2.788%]
**n6+**	11.298% [10.627%,13.205%]	13.125% [12.244%,13.813%]	12.463% [11.835%,13.511%]
**(n6-18∶2)+**	1.504% [1.302%,1.636%]	2.790% [2.273%,3.001%]	2.136% [1.952%,2.475%]

Fatty acid (FA) percentage concentrations (mol%) of plasma nonesterified fatty acids (pNEFA), subcutaneous adipose tissue (sAT) and visceral adipose tissue (vAT) are given as median and interquartile range.

*- significantly higher in vAT compared to sAT, +- significantly lower in vAT compared to sAT; Wilcoxon test adjusted for multiple testing with Sidak correction: significance is assumed with p<0.001068 achieving a global significance level of 0.05.

The most abundant FA in the studied compartments were 18∶1, 16∶0 and 18∶2, followed by 18∶0 in plasma and 16∶1 in AT.

Significant differences in FA composition between both sites of AT were mainly found for VLCFA which were higher in vAT, and some PUFA with significantly higher contribution to sAT (Table1).

**Figure 1:**
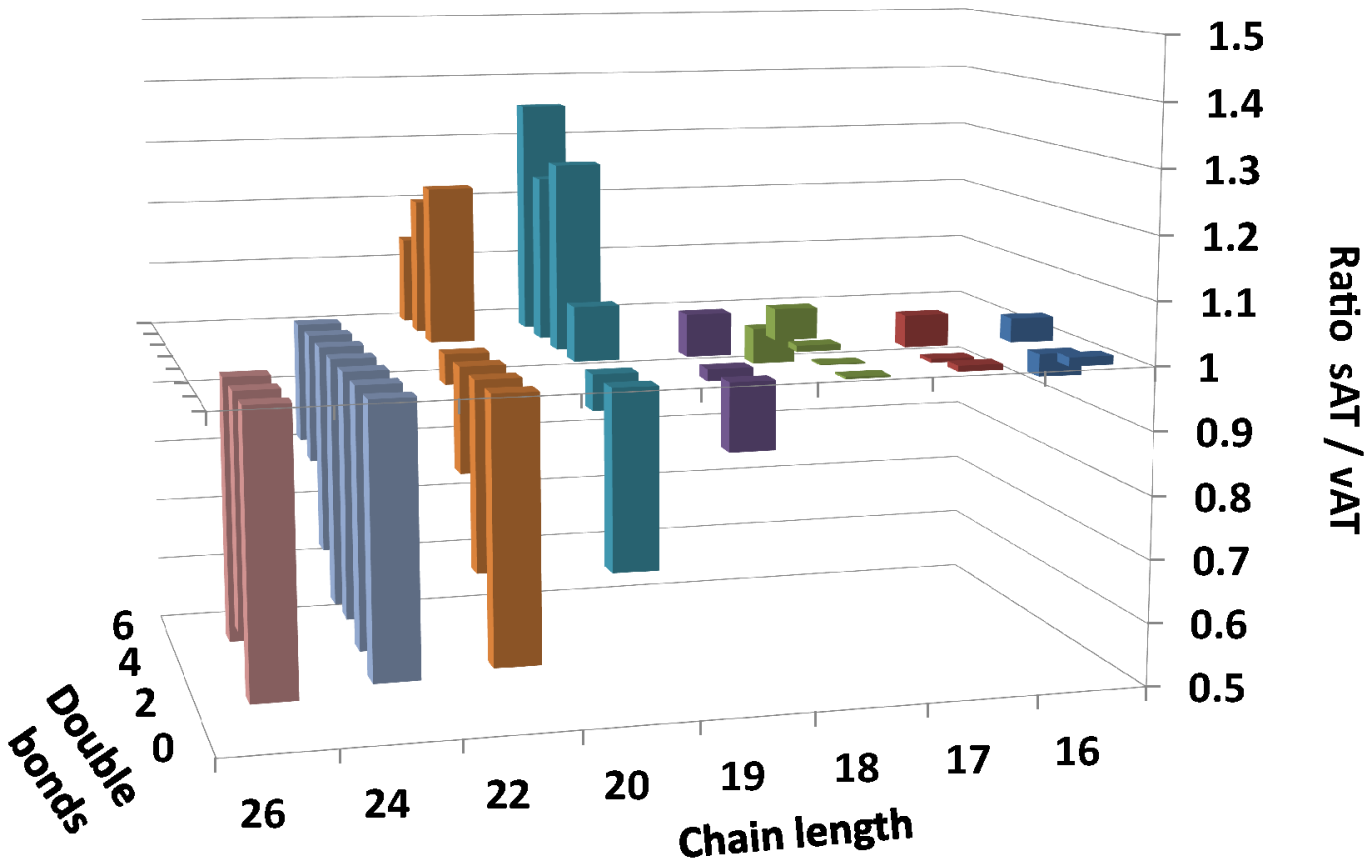
Fatty acid (FA) composition of adipose tissue sites. Ratio of FA percentages in subcutaneous adipose tissue (sAT) and visceral adipose tissue (vAT) by chain length and number of double bonds.

Plotting the ratio of sAT to vAT percentages versus carbon chain-length and number of double bonds indicated a clear trend for preferential incorporation of highly unsaturated FA into sAT, while longer carbon chain FA tended to be higher in vAT (Figure1).

No significant difference between sAT and vAT was found for medium- and long-chain SFA (14∶0,16∶0,18∶0), MUFA (14∶1,16∶1,18∶1,20∶1) and the sums of SFA and MUFA as well as odd-chain fatty acids (15∶0,17∶0,17∶1,17∶2,19∶0,19∶1,19∶2) and some PUFA.

While most FA were not significantly different between sAT and vAT, most pNEFA differed significantly from sAT, vAT or both tissues. The abundant FA 16∶0, 18∶0 and 18∶1 were significantly higher in pNEFA, compared to sAT and vAT (p<1E-07). As a consequence of the relative excess of these FA in pNEFA, percentages of most other FA were lower in pNEFA compared to AT, with two exceptions.

22∶6 had a significantly higher content in pNEFA compared to both AT, and some VLCFA (24∶1, 24∶2, 24∶3, 24∶4, 26∶2) were higher in pNEFA compared to sAT, although not statistically significant compared to vAT.

20∶4 was significantly lower in pNEFA than in sAT (p = 2.09E-4), but tended to be higher than in vAT. No significant difference was found for 18∶2, 24∶0, 26∶1 and the sum of all MUFA in pNEFA in comparison to both AT.

### Correlation of fatty acid compositions

**Figure 2:**
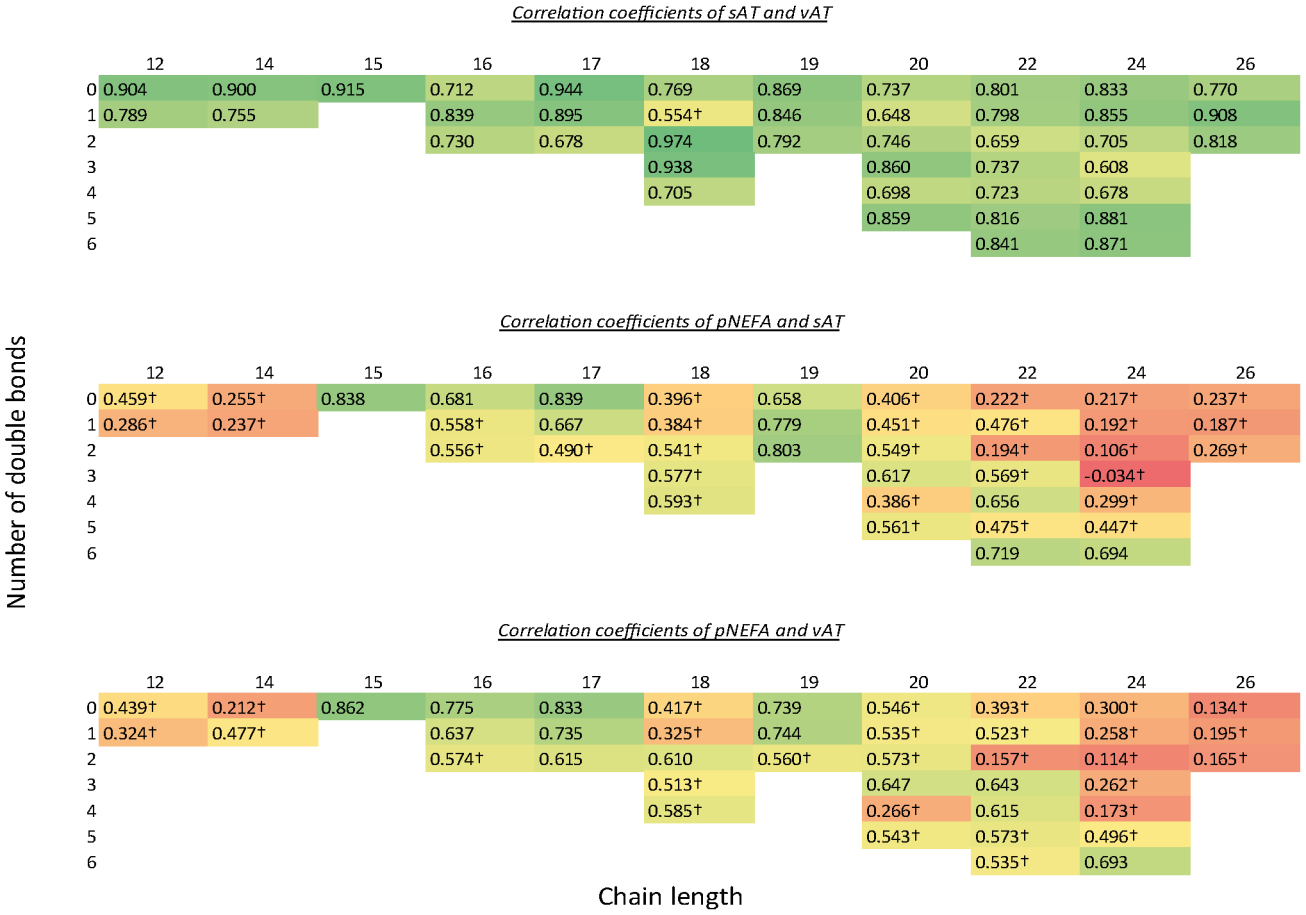
Correlation of pNEFA and AT sites. Correlation coefficients (r) of plasma nonesterified fatty acids (pNEFA), subcutaneous adipose tissue (sAT) and visceral adipose tissue (vAT). FA are arranged by chain length and double bond number. Colour gradient with red (r = 0), yellow (r = 0.5) and green (r = 1). † p>0.001068.

Most FA were closely correlated (r>0.7) between sAT and vAT, except some lower concentrated FA and 18∶1 which showed the lowest correlation coefficient (r = 0.554) (Figure2).

While most FA correlated strongly between both AT, the correlation of pNEFA and AT fatty acid percentages varied widely between FA.

16∶0 showed close correlations between pNEFA and the AT fatty acid percentages, while 16∶1, 18∶0, 18∶2 and 18∶3 had less close correlations. For the most highly concentrated 18∶1, the correlation coefficient of pNEFA with sAT was only 0.35 and 0.33 with vAT.

Odd-chain fatty acids 15∶0, 17∶0, 17∶1, 19∶0, 19∶1 and 19∶2 showed high correlation coefficients above 0.7. Also 22∶6 (r = 0.719/0.535) and 24∶6 (r = 0.694/0.693) had high correlation coefficients between pNEFA and sAT as well as vAT. In contrast, medium-chain FA correlated less between NEFA and both AT sites. Most VLCFA showed weak correlation between plasma and AT fatty acid percentages. R-values tended to increase with unsaturation, as 24∶5 (r = 0.447/0.496) and 24∶6 (r = 0.694/0.693) were the only pNEFA with more than 22 carbon atoms correlating highly with sAT and vAT.

There was a general trend for a stronger correlation of PUFA compared to their saturated and monounsaturated counterparts, except for 20∶4 which showed a low correlation to sAT (r = 0.386) and vAT (r = 0.266).

### Influence of BMI on correlation of fatty acid compositions

Quantile regression models were established for all FA analysed to determine the influence of BMI on the relation of AT fatty acid composition and pNEFA (Table2).

**Table 2:** Quantile regression models.

*FA*	*vAT*			*sAT*		
	*∼NEFA (r^2^)*	*∼NEFA+BMI (r^2^)*	*p_value*	*∼NEFA (r^2^)*	*∼NEFA+BMI (r^2^)*	*p_value*
12∶0	0.232	0.401	0.035	0.201	0.356	0.021
12∶1	0.303	0.348	0.710	0.247	0.414	0.036
14∶0	0.157	0.468	0.006	0.150	0.397	0.006
14∶1	0.437	0.492	0.013	0.221	0.316	0.229
15∶0	0.612	0.676	0.066	0.609	0.657	0.002
16∶0	0.571	0.613	0.437	0.531	0.544	0.191
16∶1	0.382	0.402	0.858	0.331	0.338	0.352
16∶2	0.426	0.504	0.176	0.291	0.285	0.555
17∶0	0.763	0.772	0.578	0.798	0.788	0.567
17∶1	0.533	0.560	0.165	0.483	0.518	0.034
17∶2	0.258	0.377	0.069	0.161	0.263	0.003
18∶0	0.304	0.528	0.085	0.203	0.289	0.246
18∶1	0.190	0.460	0.039	0.198	0.280	0.386
18∶2	0.700	0.721	0.128	0.604	0.649	0.219
18∶3	0.288	0.282	0.820	0.249	0.258	0.553
18∶4	0.320	0.490	0.191	0.453	0.527	0.246
19∶0	0.486	0.622	0.091	0.396	0.476	0.137
19∶1	0.610	0.598	0.454	0.663	0.650	0.012
19∶2	0.312	0.298	0.318	0.471	0.489	0.811
20∶0	−0.034	0.605	0.001	−0.036	0.541	0.000
20∶1	0.237	0.274	0.398	0.166	0.288	0.281
20∶2	0.386	0.284	0.576	0.465	0.449	0.891
20∶3	0.463	0.528	0.108	0.333	0.569	0.027
20∶4	−0.011	0.056	0.048	0.038	0.357	0.003
20∶5	0.225	0.242	0.147	0.230	0.351	0.182
22∶0	−0.017	0.468	0.000	−0.003	0.507	0.004
22∶1	0.280	0.462	0.000	0.244	0.383	0.168
22∶2	−0.013	0.207	0.088	0.007	0.121	0.242
22∶3	0.241	0.186	0.891	0.268	0.256	0.885
22∶4	0.661	0.677	0.550	0.634	0.625	0.669
22∶5	0.288	0.292	0.787	0.102	0.088	0.563
22∶6	0.416	0.438	0.441	0.534	0.536	0.695
24∶0	0.029	0.499	0.000	0.015	0.526	0.023
24∶1	−0.041	0.378	0.002	−0.067	0.327	0.019
24∶2	0.001	0.333	0.006	−0.015	0.197	0.318
24∶3	0.005	0.216	0.008	−0.082	−0.018	0.466
24∶4	0.087	0.247	0.174	0.143	0.221	0.643
24∶5	0.448	0.554	0.015	0.371	0.455	0.238
24∶6	0.624	0.682	0.090	0.601	0.622	0.006
26∶0	−0.001	0.466	0.000	0.000	0.461	0.002
26∶1	0.010	0.386	0.002	−0.008	0.296	0.115
26∶2	0.000	0.387	0.009	−0.011	0.303	0.073

Quantile regression models were established to predict visceral (vAT) and subcutaneous adipose tissue (sAT) by plasma nonesterified fatty acids (pNEFA)±BMI. For model comparison, ANOVA was performed whereas presented p-values indicate significant more explanation of variance with model containing pNEFA and BMI.

For some SFA, regression models including BMI in addition to pNEFA explained significantly more variance as well as in sAT and vAT. The saturated VLCFA 20∶0, 22∶0, 24∶0 and 26∶0 showed the highest relative improvement of r^2^ between models with and without BMI.

A better regression for both AT, including BMI in the regression model also appears for 12∶0, 14∶0, 15∶0, 20∶0, 20∶4 and 24∶1. Significantly more variance of vAT was explained for 14∶1, 18∶0, 18∶1, 19∶0, 22∶1, 22∶2, 24∶2, 24∶3, 24∶5 and 26∶1 by inclusion of BMI into the model. For 12∶1, 17∶1, 19∶1 and 20∶3, the inclusion of BMI improved r^2^ in sAT.

Regarding the r^2^ values for both models, the model with pNEFA only explained more than 50% of variance in both AT for 15∶0, 16∶0, 17∶0, 18∶2, 19∶1, 22∶4 and 24∶6, in sAT for 22∶6 and in vAT for 22∶6. The inclusion of BMI additionally explained more than 50% of variance in both AT for 20∶0, 20∶3 and 24∶0, in sAT for 17∶1, 18∶4 and 22∶0 and in vAT for 16∶2, 19∶0 and 24∶5.

For the strongly correlated odd-chain FA 15∶0 and 17∶0 and 22∶6, two MLR models were tested to estimate sAT and vAT fatty acid composition from pNEFA (Table3).

**Table 3:** Multiple linear regression models.

	vAT			sAT		
	Prediction error	p (model comparison)	Adjusted R-squared	Prediction error	p (model comparison)	Adjusted R-squared
						
	*MLR model 1 (pNEFA,BMI)*					
**15∶0**	1.06E-03	0.046	0.652	9.84E-04	0.073	0.629
**17∶0**	5.79E-04	0.318	0.754	4.95E-04	0.718	0.786
**22∶6**	8.10E-04	0.378	0.403	6.44E-04	0.706	0.498
						
	*MLR model 2 (pNEFA)*					
**15∶0**	1.15E-03	-	0.610	1.08E-03	-	0.595
**17∶0**	5.56E-04	-	0.753	4.70E-04	-	0.793
**22∶6**	7.95E-04	-	0.407	6.19E-04	-	0.516

Multiple linear regression models (MLR) were established to estimate fatty acid composition of subcutaneous (sAT) and visceral adipose tissue (vAT) from plasma nonesterified fatty acids (pNEFA) and/or BMI.

Validation was performed using leave-one-out cross-validation with absolute prediction errors, adjusted r squared and model comparison with ANOVA of model 1 and 2.

Both CV prediction error and adjusted r squared were similar between the models. Prediction quality was improved by including BMI only for 15∶0. ANOVA analysis showed no significant difference between the two models for 17∶0 and 22∶6, but MLR model 1 (including pNEFA & BMI) was significantly better compared to model 2 for 15∶0. However, regarding the slight difference of prediction error and adjusted R squared for 15∶0, it is also possible to establish model 2 for this FA.

**Figure 3:**
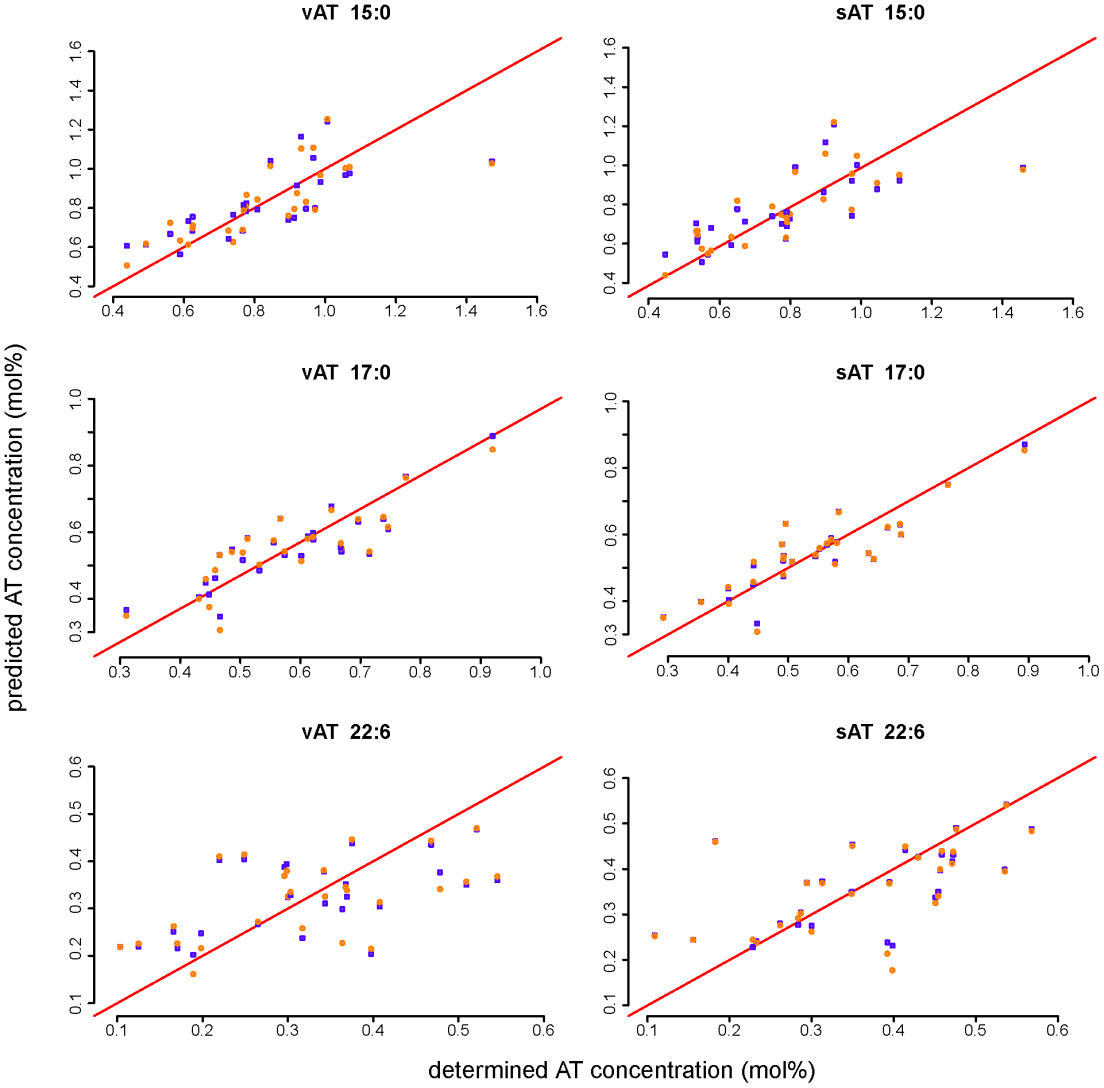
Regression models for AT FA and pNEFA. Comparison of analysed subcutaneous (sAT) and visceral adipose tissue (vAT) fatty acid composition and predicted AT fatty acid composition by using different multiple linear regression models with pNEFA (blue squares) or pNEFA+BMI (orange circles).

The strong correlation of these FA percentages in pNEFA and AT was apparent from scatter plots of AT fatty acid percentages predicted from pNEFA and actually analysed values in AT (Figure3). The values were close to the bisecting line without influence of the MLR model. This was true for correlations with sAT and vAT.

## Discussion

The present study shows a wide variation of the coefficients of correlation between fatty acid percentages of adipose tissue and nonesterified fatty acids in plasma.

Of importance, close correlations between pNEFA and AT were found for FA known to be markers of specific dietary intakes, i.e. odd-chain FA (markers for dairy fat intake) and omega3 (n-3) LC-PUFA (markers for fish intake).

The content of 15∶0 in TAG of AT is a marker for the intake of dairy products in women [27] and men [2]. Similarly, total 15∶0 in plasma and erythrocytes is used as a biomarker for dairy fat intake [28]. Nonesterified FA were not previously analysed in these or other studies on serum markers [28], [29]. Considering the strong correlation of 15∶0 and other odd-chain FA percentages in AT and pNEFA, we assume that pNEFA reflect dairy intake similar to other serum markers. Hence, 15∶0 in pNEFA could provide an alternative biomarker for habitual dairy food intake in epidemiological studies. Certain odd-chain fatty acids are also found in marine fish with up to 2.2% of 15∶0 in keeled mullet [30], while bovine milk contains about 0.9% [31].

A close correlation between dietary intake and FA percentages in AT has been well established for n-3 FA [3]. Our observation of a high correlation coefficient of 0.719 for the n-3 FA 22∶6 between pNEFA and sAT is in line with previous findings of Leaf et al. (r = 0.87) [32] and Yli-Jama et al. (r = 0.572) [9] and is further underlined by the good validation parameters of the MLR models for these FA including only pNEFA percentage as independent parameter. The similarly high correlation coefficients of 20∶5 and 24∶6 indicate a generally strong correlation of n-3 FA between AT and pNEFA. Long chain n-3 FA 20∶5 and 22∶6 share a low rate of β-oxidation in peroxisomes [17], [33]; therefore, pNEFA concentrations of individual subjects are only slightly affected by fatty acid oxidation. This agrees with the higher percentages of 22∶6 in pNEFA compared to AT.

The strong correlation of 22∶6 and other PUFA was related to higher AT mobilization rates by Yli-Jama et al. [9]. PUFA are preferentially mobilized from AT in in-vitro studies with adipocytes [12], in in-vivo studies in animals [13] and in humans [15]. The mobilization rates were not determined in the presented study, but there seems to be a general trend towards relatively high correlation coefficients for PUFA in accordance with their previously mentioned higher mobilization rates.

In contrast to other PUFA, pNEFA 20∶4 showed a low correlation with AT. This confirms the observation of Yli-Jama [9]. Low correlations for 20∶4 have also been observed between diet and AT [3], [4] as well as between glycerophospholipids from cheek cells, erythrocytes and plasma [34]. Moreover, correlation between sAT and vAT is lower for 20∶4 than for most other FA (Figure2). Although a high mobilization rate of 1.6 has been previously found for 20∶4 [12], its plasma concentration might be even stronger affected by its conversion to eicosanoids, as this fatty acid is typically the major precursor for eicosanoids [35] and 20∶4-dervied eicosanoids are formed at a faster rate than 20∶5-derived eicosanoids [18].

Another trend, contributing to the variety of the correlation pattern, was found in physicochemical properties. Increasing chain-length and increasing saturation negatively impact correlation coefficients. In previous studies, this has been ascribed to lower mobilization as well as lower clearance of SFA and MUFA, especially of 18∶0 [15]. The influence of flux to the different tissues may be confirmed by the significantly lower percentages of medium-chain FA 12∶0, 12∶1, 14∶0 and 14∶1, as these FA have a higher flux to tissue due to direct transport through cell membrane and rapid mitochondrial oxidation independently of the carnitine transport system [36].

Many metabolic processes besides AT TAG lipolysis affect pNEFA variation, such as lipolysis of lipoprotein bound TAG by LPL [37] and release or exchange of fatty acids of lipoprotein or membrane phospholipids [19]. Thus, the contribution by non AT lipolysis and cellular FA uptake for beta-oxidation and synthesis of prostaglandins may be regulated differently between FA species and individuals. As a result, the observed correlation between pNEFA and AT decreases.

Nevertheless, AT is the dominating source for pNEFA generation and mainly determines concentration as well as percentages of pNEFA, as evidenced by the similarity of molar percentages of highly concentrated FA. The close relation between pNEFA and AT is supported by strong correlation of desaturase indices [38]. High correlations for the ratio of 16∶1/16∶0, 18∶1/18∶0 (stearoyl-desaturase, respectively) and 20∶4/20∶3 (delta-5-desaturase) were found between pNEFA and sAT. The strong correlation of stearoyl-desaturase indices was confirmed in the presented study (data not shown).

In contrast to the wide variation of correlation coefficients between pNEFA and AT, correlation between sAT and vAT FA was strong for the majority of FA species, although significant differences in FA composition between the two sites of AT were found. This demonstrates that strong correlations do not necessarily depend on very similar concentrations, but stable pools with slow relative turnover rates support the establishment of close relations between tissues.

The higher amount of PUFA in sAT is in contrast to a previous study finding no significant differences of PUFA between AT sites [39]. But it agrees with the observation that subcutaneous fat is softer than deeper fat and that vAT contains more SFA compared to sAT [39].

In previous studies sAT [40], [41] or vAT [39] have been claimed to be the major source of pNEFA, while our correlation analyses did not point towards a clearly higher contribution of sAT or vAT. Remarkably, BMI has a FA specific influence on correlation. Explanation of variance of FA percentages in AT was significantly improved by including BMI additionally to pNEFA into quantile regression models only for SFA and 20∶4. Thus, with increasing BMI and increasing fat mass the correlation of SFA content between pNEFA and AT increased which may be a result of a higher contribution of FA release from AT to the pNEFA pool as well as a relatively lower flux of these pNEFA to tissue.

Eventually, the lower flux to tissue is the result of decreased β-oxidation. In obese mice lower hepatic carnitine levels were found compared to controls [42] resulting in an insufficient β-oxidation. Additionally, the lower flux to tissue and elevated pNEFA levels may result in an increased (re-)uptake of pNEFA to AT which strengthen the correlation of SFA between pNEFA and AT in obese subjects [43].

Thus, in highly obese patients a relation between SFA in pNEFA and AT FA composition can be supposed, while there is a less close relationship in normal weight subject. The findings of the present study for odd-chain FA and n-3 FA were confirmed in a correlation analysis only including the 8 normal-weight subjects (data not shown) and seem to be valid for normal weight as well as obese subjects.

A limitation of our study is that no information about habitual diet of the participants was available, as fat content of the diet may influence the specific uptake into sAT or vAT [44] and the fatty acid composition of the diet, e.g. n3/n6 ratio, affects metabolic fatty acid deposition and AT metabolism [10]. Since none of the participants followed a specific diet and they varied widely in respect to age and BMI, we can assume a wide variation of dietary preferences.

We only studied abdominal sAT, but FA uptake and composition in sAT have been found to differ between sites of sAT [45]. Abdominal sAT is most frequently collected in clinical trials and upper-body fat contributes significantly to pNEFA [14], thus, our results should be of relevance and reflect the major effects. Since our study was accomplished to determine the suitability of pNEFA as surrogate markers for AT composition, we point out that only women were studied and considering the influence of gender on sAT and vAT metabolism [46], further studies are needed to expand our results to males or infants. The strong correlation of desaturase indices in Swedish men [38] hypothesizes similar results of FA correlation in male subjects.

## Conclusion

Plasma nonesterified fatty acids are derived mainly from adipose tissue TAG hydrolysis, but additional processes, such as oxidation or prostaglandin synthesis seem to affect their flux and consequently the variation of pNEFA.

We demonstrate that nonesterified odd-chain FA in plasma reflect AT contents very well and thus seem a suitable alternative to AT biopsies for the estimation of long-term status and dietary habits. Fish intake and n-3 status of adipose tissue are reflected by pNEFA 22∶6.
